# Development of an ultra-sensitive human IL-33 biomarker assay for age-related macular degeneration and asthma drug development

**DOI:** 10.1186/s12967-021-03189-3

**Published:** 2021-12-20

**Authors:** Elaine Mai, Joyce Chan, Levina Goon, Braeden K. Ego, Jack Bevers, Tiffany Wong, Manda Wong, Racquel Corpuz, Hongkang Xi, Jia Wu, Kellen Schneider, Dhaya Seshasayee, Michele Grimbaldeston, Gerald Nakamura, Vahan B. Indjeian, Menno van Lookeren Campagne, Kelly M. Loyet, Laetitia Comps-Agrar

**Affiliations:** 1 Department of Biochemical and Cellular Pharmacology, Genentech Inc., South San Francisco, CA USA; 2 Department of Antibody Engineering, Genentech Inc., South San Francisco, CA USA; 3 Department of Structural Biology, Genentech Inc., South San Francisco, CA USA; 4 Department of OMNI-Biomarker Development, Genentech Inc., South San Francisco, CA USA; 5 Department of Immunology, Genentech Inc., South San Francisco, CA USA; 6 Present Address: Department of Biology and Compound Repository, Exelixis, Alameda, CA USA; 7 Present Address: Department of Genetics, Stanford University School of Medicine, Stanford, CA USA; 8 Present Address: Department of Inflammation and Oncology, Amgen Research, Amgen Inc., South San Francisco, CA USA

**Keywords:** IL-33, ST2, iPCR, Biomarker, Age-related macular degeneration, Asthma

## Abstract

**Background:**

Over the past decade, human Interleukin 33 (hIL-33) has emerged as a key contributor to the pathogenesis of numerous inflammatory diseases. Despite the existence of several commercial hIL-33 assays spanning multiple platform technologies, their ability to provide accurate hIL-33 concentration measurements and to differentiate between active (reduced) and inactive (oxidized) hIL-33 in various matrices remains uncertain. This is especially true for lower sample volumes, matrices with low hIL-33 concentrations, and matrices with elevated levels of soluble Interleukin 1 Receptor-Like 1 (sST2), an inactive form of ST2 that competes with membrane bound ST2 for hIL-33 binding.

**Results:**

We tested the performance of several commercially available hIL-33 detection assays in various human matrices and found that most of these assays lacked the sensitivity to accurately detect reduced hIL-33 at biologically relevant levels (sub-to-low pg/mL), especially in the presence of human sST2 (hsST2), and/or lacked sufficient target specificity. To address this, we developed and validated a sensitive and specific enzyme-linked immunosorbent assay (ELISA) capable of detecting reduced and total hIL-33 levels even in the presence of high concentrations of sST2. By incorporating the immuno-polymerase chain reaction (iPCR) platform, we further increased the sensitivity of this assay for the reduced form of hIL-33 by ~ 52-fold. Using this hIL-33 iPCR assay, we detected hIL-33 in postmortem human vitreous humor (VH) samples from donors with age-related macular degeneration (AMD) and found significantly increased hIL-33 levels when compared to control individuals. No statistically significant difference was observed in aqueous humor (AH) from AMD donors nor in plasma and nasosorption fluid (NF) from asthma patients compared to control individuals.

**Conclusions:**

Unlike existing commercial hIL-33 assays, our hIL-33 bioassays are highly sensitive and specific and can accurately quantify hIL-33 in various human clinical matrices, including those with high levels of hsST2. Our results provide a proof of concept of the utility of these assays in clinical trials targeting the hIL-33/hST2 pathway.

**Supplementary Information:**

The online version contains supplementary material available at 10.1186/s12967-021-03189-3.

## Introduction

Over the past decade, hIL-33 has emerged as a crucial immune modulator with a predominant role in infectious, fibrotic, metabolic and chronic inflammatory diseases [1, 2]. For instance, hIL-33 can be an attractive target for respiratory infection, allergy and asthma pathogenesis. Indeed, *IL33* and its Interleukin 1 receptor-like 1 (*IL1RL1*) receptor are among the most highly replicated susceptibility loci for asthma [3–5].

More recently, several reports have emerged on a role for IL-33 in ocular pathophysiology, assigning both protective [6–9] and pathogenic [10] functions to IL-33, depending on the preclinical disease model studied. We have previously reported on a role for IL-33 as a key regulator of inflammation and photoreceptor degeneration in a rodent model of retinal injury [10]. This constant light exposure (CLE) model resulted in progressive loss of rods and cones [11] with cellular and molecular features similar to the pathology observed in dry age-related macular degeneration (AMD) [12]. We demonstrated increased secretion of IL-33 by Müller cells and IL-33 dependent recruitment of pathogenic mononuclear phagocytes to the photoreceptor layer following CLE [10]. In human AMD donor eyes, we observed a higher number of hIL-33+ Müller cells and ionized calcium-binding adapter molecule 1 (Iba1) + myeloid cells in areas of retinal pigment epithelium (RPE) and photoreceptor atrophy as compared to unaffected adjacent tissues and control donor eyes [10]. Finally, IL-33 has the ability to activate mast cells [13]. Although mast cells play an important role in immunosurveillance, their dysregulation may contribute to pathogenesis as they have been shown to accumulate in the choroid of patients diagnosed with geographic atrophy (GA), an advanced form of AMD [14]. Based on these findings, we positioned hIL-33 as a potential target for AMD. As a first step towards understanding the role of hIL-33’s role in disease and pathophysiology and to validate our hypothesis, we established a sensitive assay to measure the levels of secreted hIL-33 in human VH and AH in normal versus dry AMD donors.

IL-33 exerts its cytokine activity through binding and activation of its receptor ST2 and its co-receptor IL-1 receptor accessory protein (IL-1RAcP) [15–17]. It has an elevated constitutive expression in endothelial cells, epithelial cells and fibroblast-like cells [18] and its expression can be further increased during inflammation [10, 19, 20]. IL-33 functions as an alarmin that is released upon tissue injury, cellular necrosis or mechanical stress (trauma, infection, allergens) [18, 21] to promote activation of tissue-resident immune cells expressing hST2. The full-length IL-33 that is released from the nuclei of producing cells is active. However, it can be further cleaved by inflammatory proteases that can result in the production of mature forms that exhibit 10- to 30-fold higher activity [22, 23].

Because of its potent pro-inflammatory nature [24], hIL-33 function is tightly regulated in vivo. Besides its nuclear sequestration, hIL-33 can be regulated once released from the cells by binding to the soluble form of ST2 (hsST2), which acts as a decoy receptor [25]. hsST2 originates from an alternative splice variant that lacks the sequence encoding the transmembrane domain of ST2. It is abundantly present in biological fluids and can therefore significantly dampen hIL-33 activity in vivo [25].

More recently, Cohen et al. reported another hIL-33 regulation mechanism that involves the rapid formation of two disulfide bridges within active hIL-33 in the extracellular environment [26]. Oxidized hIL-33 undergoes a conformational change that results in termination of its biological activity by preventing its binding to hST2 [26].

hIL-33 has broad implication in multiple pathologies and therefore is the target of multiple ongoing clinical trials using anti-hIL-33 antibodies (Abs). For this reason, it is crucial to develop well-characterized hIL-33 biomarker assays, as most of the information concerning the potential of hIL-33 as a target has been obtained using mouse models. The accurate measurement of active human hIL-33 in biological matrices remains a challenge for the following reasons: (1) in non to moderate pathological conditions, hIL-33 is often present at undetectable to low levels (< pg/mL), (2) hIL-33 exists in different structural forms (reduced versus oxidized) and (3) hIL-33 can be free or associated with hsST2, which can interfere with its detection [26, 27].

Here, we compared a series of commercial hIL-33 assays representing a variety of technology platforms. We then described the subsequent development and implementation of two alternative, sensitive ELISAs with equal sensitivity to reduced and oxidized forms. Because low hIL-33 concentrations can present challenges for detection by standard ELISAs, we describe a further adaptation of our reduced hIL-33 assay to the more sensitive iPCR platform. Unlike most commercially available assays, our assay can accurately quantify reduced hIL-33 in the presence of high concentrations of hsST2 in a variety of matrices. We used these assays to evaluate levels of active hIL-33 in AMD donor and asthma patient samples. We demonstrated a significant increase in reduced hIL-33 in VH from postmortem dry AMD compared to normal donor samples and, for the first time, accurately quantified the level of reduced hIL-33 in serum from normal and asthma patients. Together, these experiments provide a proof of concept for the potential utility of hIL-33 assays with sub-picomolar sensitivity to support clinical trials targeting the hIL-33/hST2 signaling pathway.

## Results

### Commercial assays failed to detect reduced hIL-33 with high sensitivity

To identify a sensitive assay capable of detecting single-digit pg/mL concentrations of reduced hIL-33 in biological matrices, we compared various commercial assay reagents and platforms that were used in the literature to report hIL-33 concentrations (Table 1). We prescreened a series of anti-hIL-33 Abs and selected only the ones yielding the highest sensitivity in buffer for testing in the ELISA format. We established four selection criteria that must be met to qualify as a suitable biomarker assay for human matrices: (1) the limit of quantitation of the assay in VH must be < 4 pg/mL as hIL-33 is expected to be absent or present at low levels under non to moderate pathology conditions, (2) the assay must tolerate at least 10 ng/mL hsST2 as hsST2 mean concentrations in VH and serum samples are 2.05 and 19.8 ng/mL, respectively (Additional file 1: Fig. S1), (3) the assay must be specific, which was characterized by the capacity to immunodeplete 100% ± 20% of hIL-33 from hIL-33-containing human VH samples when incubated with an anti-hIL-33 Ab and (4) the maximum sample volume must be 50 µL as some human ocular matrices are challenging to obtain and a high dilution would compromise the sensitivity of the assay.

**Table 1:** Performance of commercial reagents and platforms to detect hIL-33

Assay	Platform	Assay criteria				
		LLOQ in buffer (pg/mL)	LLOQ in VH (pg/mL)	sST2 tolerance (ng/mL)	Immunodepletion (%)	Sample volume (µL)
R&D Human IL-33 Quantikine Kit	ELISA	**6.25**	**62.5**	**1**	n/a	50
Biolegend LEGEND MAX™ Human IL-33 Kit		**15.6**	n/a	10	n/a	50
Invitrogen IL-33 Human Kit		1.6	3.2	**1**	81	50
Rb xhIL-33 (Pepro)/Biotin-goat xhIL-33 (R&D)		0.8	**8**	**0.25**	n/a	25
Mu xhIL-33 (Enzo)/Biotin-mu xhIL-33 (Novus)		**20**	n/a	**0.5**	**59**	25
U-PLEX Human IL-33 MSD Kit	ECL	0.6	2.4	**0.5**	**60**	25
Bio-Plex Pro™ Human Th17 Cytokine 15-Plex Kit	Luminex	1.3	2.6	**0.016**	n/a	50
Mu xhL-33 (Enzo)/Biotin-mu xhIL-33 (Novus)	Quanterix	**20**	n/a	n/a	n/a	**150**

Parameters that did not meet our assay criteria selection are highlighted in bold; n/a represents parameters not tested

None of the commercial kit/reagent assays tested met all four criteria (Table 1). The Invitrogen Human IL-33 ELISA kit, Rabbit (Rb) xhIL-33 (Pepro)/Biotin goat xhIL-33 (R&D), the U-PLEX Human IL-33 MSD kit and the Bio-Plex Pro Human T helper (Th17) cytokine 15-Plex kit had the lowest limit of quantitation in VH (Table 1). However, none of them could tolerate the mean hsST2 concentrations expected in human matrices (Additional file 1: Fig. S1). The Quanterix Simoa platform failed to improve the limit of quantitation of the mu anti-hIL-33 (Enzo)/Biotin-mu anti-hIL-33 (Novus) pair in buffer and the sample volume required was not compatible with assaying ocular samples (Table 1).

**Fig. 1:**
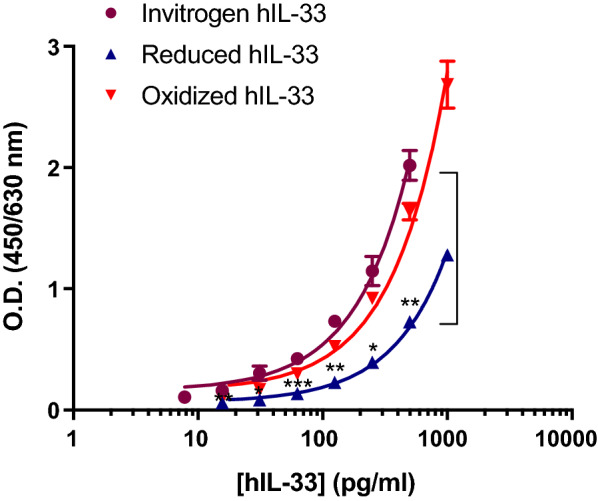
Comparison of hIL-33 standard curves established using eBioscience hIL-33 platinum ELISA kit and hIL-33 provided with the kit (purple circle), in-house reduced hIL-33 (blue triangle), and oxidized hIL-33 (red inverted triangle). Data are means ± SD of triplicates. * = P < 0.05, ** = P < 0.01, ***P < 0.001

Aside from an unacceptable hsST2 tolerance, the Invitrogen Human IL-33 ELISA kit was sensitive and specific (Table 1) and met three out of four criteria. To investigate which forms of hIL-33 were specifically recognized in this kit, we compared standard curves generated using hIL-33 provided in the kit or oxidized and reduced hIL-33 generated in house (Additional file 2: Fig. S2). Our results indicated that this ELISA kit was detecting mostly oxidized hIL-33 and has a low sensitivity towards reduced hIL-33 (Fig. 1). Altogether, our comparative study suggests that none of the tested commercial reagents/kits satisfied the criteria necessary to qualify as a sensitive reduced hIL-33 detection assay.

### Development of reduced and total hIL-33 ELISAs

**Fig. 2:**
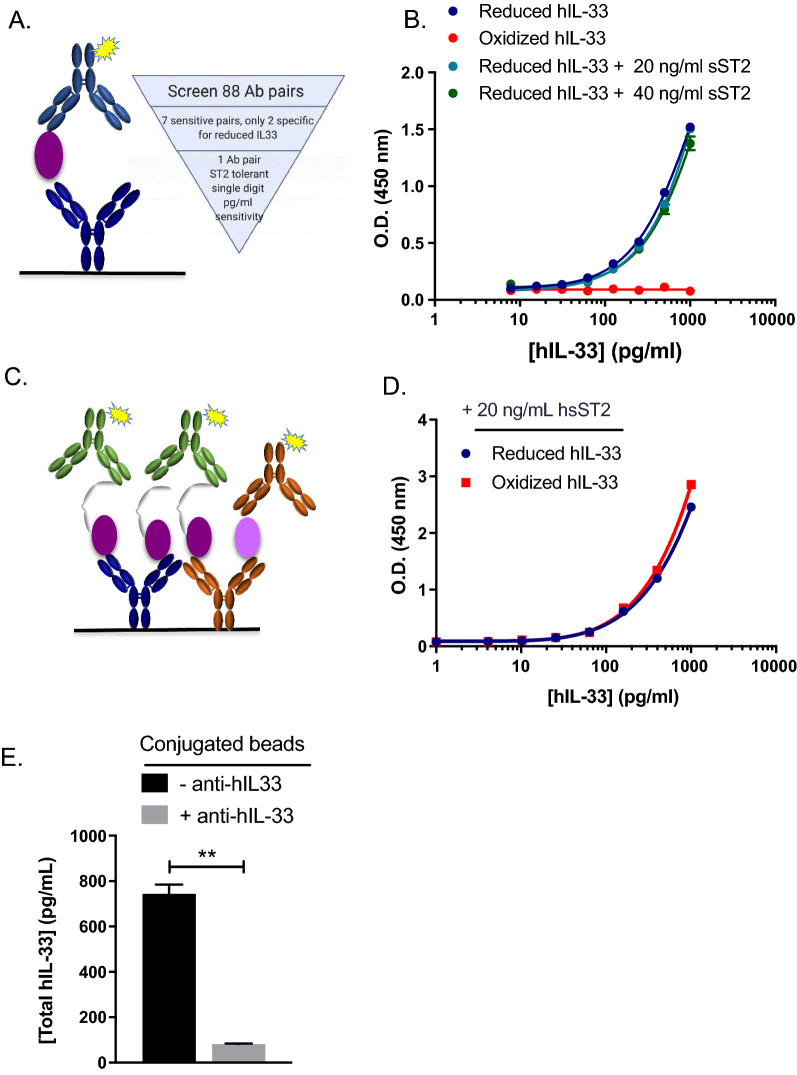
Development of reduced and total (oxidized + reduced) hIL-33 ELISAs. **A** Schematic representation of reduced hIL-33 ELISA format: in-house rat anti-hIL-33 15.21C7 capture MAb (dark blue), in-house reduced hIL-33 standard (purple), and in-house biotinylated rat/human chimeric anti-hIL-33 3F10 detection MAb (light blue). **B** Comparison of standard curves obtained with reduced hIL-33 (blue), oxidized hIL-33 (red), reduced hIL-33 + 20 ng/mL hsST2 (light blue), reduced hIL-33 + 40 ng/mL hsST2 (green). **C** Schematic representation of total hIL-33 ELISA format: in-house rat anti-hIL-33 15.21C7 capture MAb (dark blue) and R&D System’s goat anti-hIL-33 PAb (orange), in-house reduced hIL-33 (purple) and oxidized hIL-33 (light purple) standards in the presence of hsST2 (gray), and R&D System’s biotinylated goat anti-hST2 (green) and goat anti-hIL-33 (orange) PAbs. **D** Comparison of reduced and oxidized hIL-33 standard curves in presence of 20 ng/mL hsST2. Data are representative of three experiments performed in duplicates. **E** Specificity of total hIL-33 ELISA determined by immunodepletion of reduced and oxidized hIL-33 in diluted human VH samples using beads conjugated to hIL-33 Ab. P = 0.0019. Data are means ± SD of duplicates

The successful development of two ELISAs capable of quantifying (1) reduced hIL-33 and (2) total hIL-33 (reduced and oxidized) in VH was accomplished through an extensive Ab generation and screening campaign followed by characterization and validation experiments. The rat immunization campaign resulted in the generation of 3026 Abs. We utilized array-based Surface Plasmon Resonance (SPR) to identify purified hybridoma Abs that specifically bound to reduced hIL-33 with high affinity and represented different epitope groups that were non competing with hsST2 through Ab binning. We then screened 88 combinations of the selected in-house monoclonal and commercial polyclonal Abs (MAbs or PAbs) pairs for their ability to bind total, oxidized or reduced hIL-33 by ELISA. Out of the 88 Abs pairs, two detected reduced hIL-33 only with single-digit pg/mL sensitivity in buffer and only one pair (Ab clones 3F10 and 15.21C7) was hsST2 tolerant (up to 40 ng/mL) (Fig. 2A, B). Clones 3F10 and 15.21C7 bound reduced hIL-33 with constant dissociation (K_D_) values of 0.487 and 0.338 nM, respectively (Additional file 3: Fig. S3). The lower limit of quantitation (LLOQ) and the upper limit of quantitation (ULOQ) of the reduced hIL-33 ELISA were 4.1 pg/mL and 1000 pg/mL, respectively with inter-assay coefficient of variations (CVs) of 9.1% and 1.6% (Table 2). The assay was performed in 384 well plate format with an assay volume of 25 µL.

**Table 2:** Standard curves performance of reduced and total hIL-33 ELISA

	Nominal conc (pg/mL)	Measured mean conc (pg/mL)	% Recovery	% CV
Reduced hIL-33 ELISA	1000	991.5	99.2	1.6
	400	408.2	102.1	2.8
	160	158.5	99.0	1.7
	64	63.6	99.4	1.5
	25.6	26.2	102.3	7.0
	10.2	11.0	107.6	5.6
	4.10	3.83	93.6	9.1
Total hIL-33 ELISA	1000	1007.4	100.7	0.7
	400	407.5	101.9	2.5
	160	159.6	99.7	2.4
	64	64.7	101.1	8.4
	25.6	27.6	107.9	8.4
	10.2	9.56	93.4	9.6
	4.10	4.54	110.8	10.5

n = 6 independent assay runs. LLOQ = 4.1 pg/mL, ULOQ = 1000 pg/mL

We then implemented a total hIL-33 ELISA, in which we used a combination of reduced (15.21C7) and total hIL-33 specific Abs as a coat. Upon addition of a saturating concentration of hsST2, we used an Ab cocktail consisting of anti-hIL-33 and anti-ST2 MAbs to detect the totality of reduced and oxidized hIL-33 present in the mixture (Fig. 2C). The ratio of each Ab was extensively optimized to ensure equal detection of reduced and oxidized hIL-33 (Fig. 2D). The LLOQ and ULOQ of the total ELISA was 4.1 pg/mL and 1000 pg/mL, respectively, with inter-assay CVs of 10.5% and 0.7% (Table 2).

Immunodepletion experiments confirmed that the assay was specific for total hIL-33 in control VH samples with a reduction of 89.1% ± 0.31% signal in the presence of beads conjugated anti-hIL-33 Abs (Fig. 2E). We also established the minimum required dilution to be 1:2 for serum, plasma, and NF by testing total hIL-33 linearity dilution (Additional file 4: Table S1). As the LLOQ of the assay is 4.1 pg/mL, the lowest limit of detection is 8.2 pg/mL for serum, plasma and NF.

To summarize, we have successfully implemented and developed a reduced and total hIL-33 ELISA that can detect single-digit pg/mL hIL-33 in the presence of hsST2.

### Development and characterization of reduced hIL-33 iPCR assay

**Fig. 3:**
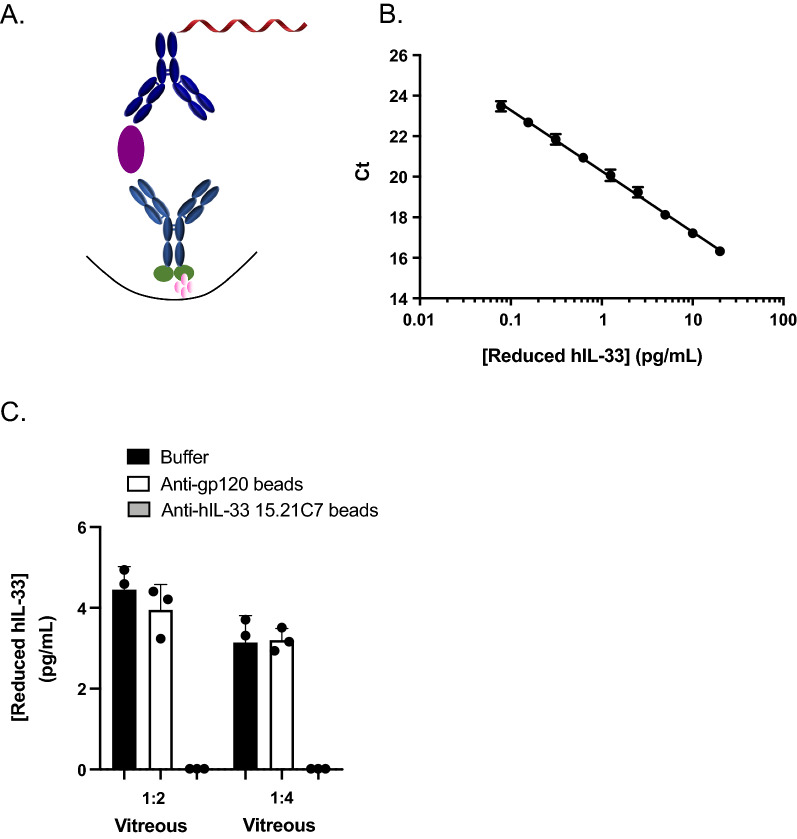
Development and specificity of 384-well reduced hIL-33 immuno-PCR (iPCR) assay. **A** Schematic representation of reduced hIL-33 iPCR assay format: biotinylated rat/human chimeric anti-hIL-33 3F10 capture MAb (light blue), reduced hIL-33 standard (purple), and 55-mer DNA-labeled rat IgG2a anti-hIL-33 15.21C7 detection MAb (dark blue). **B** Reduced hIL-33 standard curve obtained using iPCR in sample buffer. Data are means ± SD of 6 independent standard curves, each performed with 4 replicates. **C** Specificity of reduced hIL-33 iPCR assay determined by immunodepletion of reduced hIL-33 in diluted human VH samples using beads conjugated to rat anti-reduced hIL-33 15.21C7 IgG2a (gray bars), control beads conjugated to rat anti-gp120 IgG2a (white bars) and sample buffer (black bars). P = 0.0004 (1:2 VH), P = < 0.0001 (1:4 VH). Data are means ± SD of triplicates

Despite the increased sensitivity of our in-house hIL-33 ELISA, it still failed to measure detectable levels of hIL-33 in a large proportion of human ocular samples and serum samples (data not shown). Therefore, we sought to investigate the iPCR platform that had previously showed great promise in increasing the sensitivity of detection assays [28–31]. Similar to the reduced hIL-33 ELISA, we utilized the clones 3F10 and 15.21C7 but conjugated the latter to a 55-mer DNA that was amplified during the iPCR reaction, allowing for signal intensification (Fig. 3A). The iPCR platform dramatically increased sensitivity with a LLOQ of 0.078 pg/mL (± 1.1%), representing a 52.6-fold improvement from the ELISA (Table 3, Fig. 3B). Immunodepletion experiments confirmed that the assay was specific for reduced hIL-33 in control VH samples with a reduction of 99.6% ± 0.07% signal in the presence of beads conjugated to clone 15.21C7 Ab (Fig. 3C). Of note, we were unable to successfully transfer our total hIL-33 ELISA to the iPCR platform (data not shown).

**Table 3:** Standard curve performance of reduced hIL-33 iPCR

Nominal conc (pg/mL)	Measured mean conc (pg/mL)	% Recovery	%CV
20	19.06	95.3	1.5
10	9.93	99.3	1.2
5	5.18	103.6	1.2
2.5	2.32	92.9	1.3
1.25	1.24	98.9	1.5
0.63	0.63	101.0	0.9
0.31	0.31	98.2	1.2
0.16	0.15	97.3	0.9
0.078	0.074	94.8	1.1

n = 6 independent assay runs. LLOQ = 0.078 pg/mL, ULOQ = 20 pg/mL

We evaluated the assay precision and our three diluted reduced hIL-33 QC controls had intra- and inter-assay CVs lower than 20% (Table 4), showing that the results generated were reproducible and precise.

**Table 4:** Precision of reduced hIL-33 iPCR assay

Control	Mean (pg/mL)	%CV	
		Inter-assay^a^	Intra-assay^b^
High control	6.52	15	9.4
Mid control	1.82	6.9	13
Low control	0.591	13	8.7

^a^n = 12 runs

^b^n = 4 replicates

We then assessed the assay linearity using a set of pooled, individual control and diseased matrix samples in order to determine the minimum required dilution. As we were interested in implementing a biomarker assay that can be used across multiple indications, we tested two ocular matrices, serum, plasma, and NF. Due to low endogenous reduced hIL-33 concentrations (low pg/mL) and limited availability of human matrices, the range of dilutions tested was reduced. Our results showed that the recoveries among dilution-adjusted concentrations of ocular, serum, plasma and NF matrices were within the ± 20% acceptance criterion for linearity across all dilutions tested except for serum at 1:2 (% recovery = 66.3%) (Table 5). Therefore, the minimum required dilution (MRD) for VH, AH, plasma and NF is 1:2 and 1:4 for serum. As the LLOQ of the assay is 0.078 pg/mL, the lowest concentrations that can be quantitated were 0.156 pg/mL for VH, AH, plasma and NF, and 0.312 pg/mL for serum.

**Table 5:** Dilution linearity of reduced hIL-33 endogenously expressed in human matrices

Dilution factor in matrix	Observed (pg/mL)	Corrected (pg/mL)	% Recovery
1:2 VH	3.44	6.87	110.7
1:4 VH	1.45	5.78	93.1
1:8 VH	0.75	5.98	96.2
1:2 AH	3.21	6.43	98.3
1:4 AH	1.66	6.65	101.7
1:2 serum	3.02	6.04	66.3
1:4 serum	2.24	8.94	98.2
1:10 serum	1.07	10.70	117.5
1:20 serum	0.54	10.75	118.0
1:2 plasma	4.77	9.55	96.4
1:4 plasma	2.79	11.14	112.5
1:10 plasma	1.03	10.27	103.7
1:20 plasma	0.43	8.64	87.3
1:2 NF	7.91	15.82	109.1
1:4 NF	3.94	15.77	108.7
1:10 NF	1.32	13.16	90.7
1:20 NF	0.66	13.29	91.6

Additionally, we performed spike recovery experiments to evaluate accuracy. All sample recovery was in the expected 80–120% range, demonstrating that the iPCR assay can accurately measure reduced hIL-33 in ocular, serum, plasma and NF matrices (Table 6).

**Table 6:** Accuracy test as measured by spike recovery of reduced hIL-33 in matrices using reduced hIL-33 iPCR assay

hIL-33 (pg/mL)	VH (1:2)	AH (1:2)	Serum (1:4)	Plasma (1:2)	NF (1:2)
Endogenous conc	< 0.078	0.59	2.24	5.73	7.91
Spiked conc. (low)	0.47	0.47	0.45	0.36	0.45
Measured conc	0.41	1.14	3.14	7.26	9.78
Expected conc. (endo + spike)	0.47	1.06	2.69	6.09	8.36
% Recovery	86.5	107.1	117.0	119.2	117.0
Endogenous conc	< 0.078	0.59	2.24	4.78	7.91
Spiked conc. (mid)	1.11	1.36	1.51	1.40	1.51
Measured conc	0.93	1.73	3.48	5.90	11.0
Expected conc. (endo + spike)	1.11	1.95	3.75	6.18	9.42
% Recovery	84.1	88.7	92.8	95.5	116.8
Endogenous conc	< 0.078	3.21	2.24	5.73	7.91
Spiked conc. (high)	4.25	4.25	4.48	5.22	4.48
Measured conc	3.49	6.9	5.44	10.48	14.7
Expected conc. (endo + spike)	4.25	7.47	6.72	10.95	12.39
% Recovery	82.1	92.4	81.0	95.8	118.3

### Reduced hIL-33 levels are significantly higher in VH samples from human dry AMD as compared to control

**Fig. 4:**
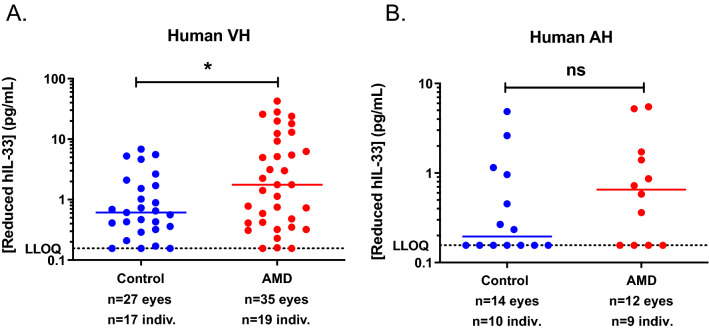
Reduced hIL-33 concentrations in VH and AH samples measured by reduced hIL-33 iPCR assay. **A** Reduced hIL-33 concentrations of postmortem human VH samples from 17 control (blue circles) and 19 AMD (red circles) donors. P = 0.0155. **B** Reduced hIL-33 concentrations of postmortem human AH samples from ten control (blue circles) and nine AMD (red circles) donors. ns = no significance. Data are means of duplicates

Using our iPCR assay, we measured reduced hIL-33 levels in human VH and AH samples from postmortem normal and AMD donors. It should be noted that information regarding the severity (early versus advanced) or type (wet versus dry) of AMD from these donors was not available. A total of 88.7% of VH and 57.7% of AH samples were above LLOQ. We observed a significant increase of reduced hIL-33 in AMD versus control samples, consistent with increased presence of hIL-33 positive cells associated with GA [10] (Fig. 4A). We also detected an average of 3.3-fold higher amounts of reduced hIL-33 in postmortem AMD AH samples as compared to control AH samples but the difference was not significant (P = 0.2773) (Fig. 4B). Altogether, our results suggest that the reduced iPCR assay can be used as a predictive and pharmacodynamic biomarker assay.

### Reduced hIL-33 is not significantly upregulated in plasma and NF from self-reported asthma patients

**Fig. 5:**
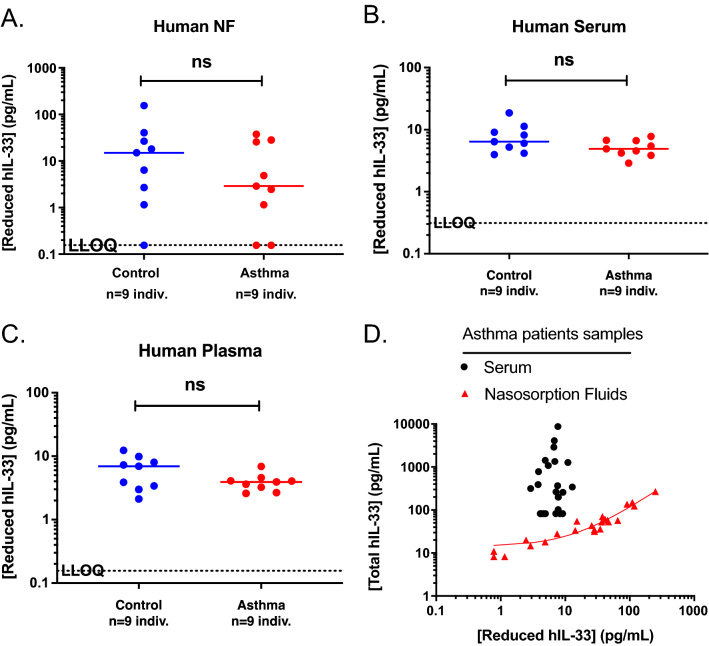
Reduced hIL-33 concentrations in serum, plasma, and NF samples measured using the reduced hIL-33 iPCR assay. **A**–**C** Reduced hIL-33 concentrations measured in human NF (**A**), serum (**B**) and plasma samples (**C**) from nine control (blue circles) and nine asthma (red circles) patients. *ns* no significance. **D** Correlation of total versus reduced hIL-33 concentrations in serum (black circles) and NF (black triangles) samples from asthmatic donors (n = 23). R^2^ = 0.95, P < 0.0001 for NF. Data are means of duplicates or quadruplicates

Several reports in the literature indicate that hIL-33 protein level is upregulated in asthma patients [32–35]. However, our comprehensive evaluation of the commercial assays available to measure hIL-33 concentrations revealed that most if not all of them are unable to measure active reduced hIL-33 with the sensitivity required to make an informed interpretation. Here, using our reduced hIL-33 iPCR assay, we determined and compared levels of reduced hIL-33 in NF and serum from nine age- and gender-matched control and asthma patients. Note that both matrices were drawn from the same donors. Our results indicate that there was no significant difference between reduced hIL-33 concentrations in NF and serum (Fig. 5A, B). In addition, the mean level of reduced hIL-33 in serum was 6.4 pg/mL (Fig. 5B). One can argue that this relatively high level can be the result of the release of intracellular hIL-33 from the platelets during serum preparation. To rule out this hypothesis, we performed similar measurements in the matched plasma samples. The mean reduced hIL-33 concentration was similar to serum (6.9 pg/mL), confirming that the reduced hIL-33 concentration is not an artefact of samples preparation (Fig. 5C). We also tested the total hIL-33 concentrations (reduced and oxidized) in the same samples and did not observe significant differences between normal and asthma samples (Additional file 5: Fig. S4).

The average reduced hIL-33 concentration in human serum from asthma patients (n = 23 samples) was 6.7 pg/mL versus 1.06 ng/mL total hIL-33, demonstrating that most of the hIL-33 was oxidized and inactive (Fig. 4D). There was a significant correlation between reduced and total hIL-33 in NF samples, demonstrating that the majority of hIL-33 in this matrix is reduced and active (Fig. 4D).

Altogether, our results do not support an upregulation of active hIL-33 in asthma patient serum and NF.

## Discussion

In this study, we provide an extensive evaluation of commercially available assays to measure hIL-33 in biological matrices. Similar to the observations made by Cohen et al. and Ketelaar et al., we confirmed that all the commercial assays we tested did not meet our criteria for sensitive and specific measurement of active secreted hIL-33 [26, 27]. This finding suggests that the bioactive hIL-33 levels in extracellular matrices reported in the literature should be interpreted with caution and supports the need to implement an assay to measure the availability of reduced hIL-33 in normal and diseased human patients.

Here, we report the implementation of an in-house, quantitative ultra-sensitive iPCR assay compatible with a 384-well plate format that accurately measures reduced hIL-33 in ocular matrices, serum, plasma, and NF with a limit of detection of 0.156, 0.312, 0.156 and 0.156 pg/mL, respectively. We chose the iPCR platform, first described by Sano et al. [28], as it has been successfully utilized to enhance detection sensitivity of antigens up to 2 logs as compared to standard ELISA [29–31]. The 384-well plate format was especially attractive to us, as human ocular samples are extremely challenging to acquire, and typically less than 100 µL of AH sample can be obtained per donor. Our fully characterized assay has the potential to be utilized as a predictive or pharmacodynamic biomarker assay.

We first measured levels of hIL-33 in human VH and AH samples from normal and AMD patients. Contrary to rodent ocular samples that contain ng/mL of hIL-33 (data not shown), we were expecting ~ 1000-fold less hIL-33 in human ocular samples based on the work published by Takeuchi et al. [36]. In this study, the authors reported a mean of 17.1 pg/mL of hIL-33 in VH samples from patients with proliferative diabetic retinopathy with only 25% of the samples above the limit of detection. Our results showed that the assay used in the study was suboptimal and could not tolerate ng/mL concentrations of hsST2 (Fig. 1). Using our iPCR assay, we were able to detect hIL-33 in 88.7% of the VH samples tested (control and AMD samples). We observed a significant increase in hIL-33 concentrations in postmortem AMD compared to control VH samples, suggesting that hIL-33 may be upregulated in AMD, consistent with earlier findings by our group [10]. We also measured an increased level of hIL-33 in postmortem AH samples from donors with AMD compared to control donors, but the difference was not significant (P = 0.2773). A higher number of AH samples may be required to establish whether a statistical significance can be reached. Overall, the hIL-33 concentrations obtained in VH samples were higher than in AH samples. This is in agreement with the predominant production of hIL-33 by cells in the posterior compartment of the eye, which is closer to the vitreous compartment than to the aqueous compartment [10].

We also analyzed serum, plasma, and NF from normal versus asthma patients. We observed a strong correlation between reduced and total hIL-33 in the NF of asthmatic patients, indicating that most of the total hIL-33 is reduced, which may be explained by the proximity of a tissue expressing high levels of hIL-33.

In contrast, most of the hIL-33 in serum and plasma seems to be oxidized and therefore unable to bind hST2. This observation confirms the prediction made by Cohen et al., who hypothesized that any free hIL-33 reaching the plasma would likely be oxidized within a few hours [26]. This regulatory mechanism is essential, as persistent elevation of IL-33 in serum has been shown to trigger lethal inflammation in a mouse model in which IL-33 nuclear localization signal was deleted [24].

Importantly, our reduced hIL-33 iPCR assay is highly hsST2 tolerant, meaning that we cannot discriminate between free reduced hIL-33 and hsST2-bound hIL-33. It implies that the 6.9 pg/mL (0.38 pM) of hIL-33 measured in plasma may not be fully active, as a fraction may be bound to its decoy receptor hsST2. We determined that the mean concentration of hsST2 in both control and asthma patients’ samples groups was 14.7 ng/mL (0.4 nM) (Additional file 6: Fig. S5A) therefore it is likely that the majority or the entire reduced hIL-33 was in complex with hsST2 and unable to signal through its receptor. We cannot make a similar assumption for the NF samples as hsST2 levels were below the LLOQ in about 60% of the samples (Additional file 6: Fig. S5B).

To our knowledge, this is the first study accurately reporting reduced and total hIL-33 in human serum and plasma. Other groups reported levels of serum hIL-33 across asthma patients with variable disease severity [32–35]. All used commercial kits to generate hIL-33 levels that, according to our thorough characterization, were not suitable to accurately detect reduced hIL-33 for the following reasons: (1) these assays could not tolerate the hsST2 level present in serum (~ 20 ng/mL), (2) their limit of detection was not low enough and/or (3) they specifically detected the oxidized form of hIL-33 only. Therefore, caution should be taken when comparing the data generated with these assays.

Our results do not show a significant difference between reduced hIL-33 levels from normal and asthmatic patients. One important point to consider is that the samples we analyzed were collected from “self-reported” asthmatics, which is likely representative of a heterogeneous population across a range of severities. We do not have information on whether these donors were undergoing asthma exacerbation at the time of sample collection, so we cannot rule out that reduced hIL-33 would indeed be significantly higher in these instances. It would also be interesting to test sputum samples from moderate-to-severe asthmatics, similarly to Cohen et al., as the human lung tissue contained high levels of hIL-33 [26].

## Conclusions

In conclusion, commercially available methods to measure reduced hIL-33 concentrations in multiple human matrices did not meet our criteria for sensitivity and specificity. To address this, we developed an in-house, sensitive iPCR assay that can specifically and accurately detect reduced hIL-33 across a wide range of matrices. This novel biomarker assay may have broad implications for ongoing and future clinical trials targeting the hIL-33/hST2 signaling pathway for multiple indications. In a clinical context, it may help identify potential responders based on basal hIL-33 concentrations and determine target engagement for patients receiving anti-hIL-33 Ab treatment.

## Materials and methods

### Measurement of human sST2 levels

hsST2 levels were determined using the Human ST2/hIL-33R Quantikine kit ELISA per the manufacturer’s instructions (#DST200, R&D Systems, Minneapolis, MN, USA). The hsST2 standard range is 7.81 pg/mL to 1000 pg/mL with a sensitivity of 15.6 pg/mL in buffer and a lowest detectable concentration of 1563 pg/mL in AH (minimum required dilution (MRD) is 1:100), 625 pg/mL in VH (MRD is 1:40), 2500 pg/mL in serum (MRD is 1:160), and 1250 pg/mL in NF (MRD is 1:80).

### Measurement of hIL-33 levels in VH using commercial kits

The following kits were used according to manufacturer’s instructions: Human IL-33 Quantikine kit ELISA (#D3300B, R&D Systems, Minneapolis, MN, USA), LEGEND MAX™ hIL-33 kit ELISA (#435907, Biolegend, San Diego, CA, USA), Invitrogen IL-33 Human ELISA kit (#BMS2048, ThermoFisher Scientific, Carlsbad, CA, USA), U-PLEX hIL-33 assay (#K151WFK, MSD, Rockville, MD, USA), Bio-Plex Pro™ Human Th17 Cytokine 15-Plex Luminex assay (#171AA001M, Bio-Rad, Hercules, CA, USA).

In addition, 2 ELISAs were developed using commercial antibodies (Abs). For the first assay, 2 µg/mL of mouse anti-hIL-33 diluted in 0.05 M sodium carbonate buffer pH 9.6 (#ALX-804-840PF-C100, Enzo, Farmingdale, NY, USA) was used to coat MaxiSorp plates (384-well, Nunc, Thermo Fisher Scientific, Rochester, NY, USA) overnight at 4 °C. After 3 washes in 0.05% Tween 20 in PBS, pH 7.4, plates were blocked using 0.5% bovine serum albumin (BSA), 15 ppm ProClin 300 in PBS, pH 7.4 for 1 h (h) at room temperature (RT). After 3 washes, titrated hIL-33 was added to the plates and incubated for 2 h at RT. The plates were washed 6 times and biotinylated goat anti-hIL-33 (#BAF3625, R&D Systems, Minneapolis, MN, USA) diluted at 1:1000 was incubated for 1 h at RT. Bound Abs were detected using streptavidin Poly-horseradish peroxidase (HRP)80 (#65R-5119, Fitzgerald, Acton, MA, USA) and its substrate 3,30,5,50-tetramethyl benzidine (Moss Inc., Pasadena, MD, USA). The reaction was stopped with 1M phosphoric acid and absorbance was read at 450 nm.

For the second ELISA, a similar protocol was followed with the exceptions that 2 µg/mL of rabbit anti-hIL-33 (#500-P261, Peprotech, Minneapolis, MN, USA) was used for coating the plate and 50 ng/mL of biotinylated mouse anti-hIL-33 clone 6H617 (#NBP2-273338, Novus, Centennial, CO, USA) was used for detection in combination with streptavidin HRP (#RPN1231, GE Healthcare Life Sciences, Chicago, IL, USA).

### Characterization of commercial and in-house hIL-33 assays in human matrices

#### Sensitivity

The lower and upper limits of quantitation (LLOQ and ULOQ) were defined as the lowest and highest hIL-33 concentrations that can be accurately quantitated. These values were determined from standard curves run in quadruplicates in 6 independent assays. The CVs and relative error (RE) were calculated for each point of the standard curves and the ones with a CV and RE below 20% were included in the reportable range.

#### Dilutional linearity

Serum, plasma and NF samples from spike recovery experiment were diluted in assay buffer at 1:2, 1:4, 1:10, and 1:20 concentrations, whereas VH and AH were diluted at 1:2, 1:4 and 1:8 (for VH only) due to limited sample volume availability. The percent recovery from measured versus expected corrected concentrations was calculated.

The MRD was defined as the minimum required dilution for which the reduced hIL-33 concentration was between 80–120% of expected sample recovery. The LLOQ in matrices was calculated by multiplying the LLOQ obtained in buffer by the MRD [37].

#### Accuracy (spike recovery)

Reduced hIL-33 was spiked into serum, plasma, and NF samples from different individuals and into pooled VH and AH samples from 2 individuals (3 to 4 replicates) to target high (4.5 pg/mL), mid (1.5 pg/mL), and low (0.5 pg/mL) concentrations. The same high, mid, and low concentrations were spiked into assay buffer as controls (Spiked concentration). Endogenous levels of reduced hIL-33 were determined from unspiked matrices. Spike recovery was calculated using the following formula:

$$\% {\text{ Recovery}} = \left[ {{\text{Measured concentration}}/{\text{Expected concentration}}\left( {{\text{endogenous}} + {\text{spike}}} \right)} \right] \times {1}00.$$

#### hsST2 tolerance determination

hsST2 tolerance was determined by spiking various concentrations of hsST2 (0.016–40 ng/mL) into hIL-33 standard curves. We reported the highest concentration of hsST2 for which an acceptable recovery of hIL-33 concentration was obtained. An acceptable recovery was defined as 100% ± 20% of the corresponding hIL-33 concentration measured in the absence of hsST2.

#### hIL-33 immunodepletion

Immunodepletion was performed by adding buffer, biotinylated anti-hIL-33 conjugated beads or anti-gp120 conjugated beads (isotype control) to human VH samples for 1 h at RT. After incubation, the conjugated beads were removed from the matrix. These two steps were repeated and the amount of hIL-33 immunodepleted from the matrix was calculated. The immunodepletion was considered acceptable if the quantity of hIL-33 in the sample was reduced by 100% ± 20% SD compared to the control (non-immunodepleted) matrix sample value.

#### Assay precision

Intra-assay precision was determined by measuring three QC samples consisting of 0.6, 1.8 and 6.5 pg/mL of reduced hIL-33 diluted in assay buffer in quadruplicates in a single run, whereas inter-assay precision was determined by measuring the 3 QC samples for a total of 12 independent runs. The assay precision is considered acceptable if the intra- and inter-assay CVs are lower than 20%.

### Reduced and oxidized hIL-33 production and quality control (QC)

A recombinant hIL-33 consisting of an N-terminal sequence MHHHHHHGENLYFQG (His-tag and TEV cleavage site) followed by the coding sequence for amino acids 112–270 (UniProtKB/Swiss-Prot Accession number 095760) was expressed in *Escherichia coli*. The protein was purified over a Ni-Excel chromatography followed by a purification on the Superdex S75 size exclusion column pre-equilibrated with PBS (phosphate buffered saline). Mass spectrometry indicated that purified hIL-33 had the expected molecular weight and amino acid sequence. From an examination of sodium dodecyl sulfate polyacrylamide (SDS-PAGE) gels, the purity of the material was estimated to be greater than 95%. An aliquot of the purified hIL-33 was further diluted into PBS buffer to obtain a final concentration of 0.3 mg/mL. Similarly, another aliquot of the purified IL-33 was diluted into 60% Iscove’s Modified Dulbecco’s Medium (IMDM) (Thermo Fischer Scientific, Waltham, MA) to obtain the same final protein concentration. The diluted proteins were then incubated at 37 °C for approximately 18 h. Both diluted proteins were then analyzed on SDS-PAGE gel and using mass spectrometry to determine the extent to disulfide bond formation. The oxidized hIL-33 diluted into the IMDM media was subsequently buffer exchanged into the final PBS buffer to remove the media components. Reduced hIL-33 in PBS was supplemented with 0.1 mM Tris (2-carboxyethyl)phosphine (TCEP) reducing agent.

### Ab production

Nine Sprague Dawley rats (Charles River, Hollister, CA, USA) were immunized with hIL-33 (Genentech, PUR36805) at 100 µg/animal for the first dose and 50 µg/animal for the rest of the boosts, each divided among sites: intraperitoneal, s.c. (subcutaneous) at base of tail, s.c. at nape of neck, and s.c. in both hocks. A Toll-like receptor cocktail including monophosphoryl-lipid A (MPL) (Sigma-Aldrich, St. Louis, MO, USA), Poly (I:C), R848, and cytosine phosphodiester bond guanine (CpG) (InvivoGen, San Diego, CA, USA) was used as adjuvant for the first dose. Antigen-specific hybridomas were generated, sorted and screened as described [38]. Briefly, Immunoglobulin G (IgG)^+^ hIL-33^+^ hybridomas were single cell sorted using hIL-33 conjugated to PE (Novus Biological, Littleton, CO, USA) into 96-well plates and cultured for 7 days. Supernatants were screened by high throughput (HTP) ELISA using robotic platforms and hybridomas showing binding to hIL-33 by ELISA were scaled-up in 1 mL cultures. Supernatants were harvested and purified using a protein G affinity chromatography resin (GammaBind Plus, GE Healthcare, Pittsburgh, PA, USA). Anti-hIL-33 Abs were submitted for sequencing and cloned out for recombinant production.

### Characterization of anti-hIL-33 Abs by array-based SPR imaging system

An array-based SPR imaging system (CFM/IBIS, Carterra, Dublin, CA, USA) was used to analyze binding kinetics and epitope bin a panel of anti-hIL-33 MAbs. Purified hybridoma Abs were diluted at 10 µg/mL in 10 mM sodium acetate buffer pH 4.5. Using amine coupling, Abs were directly immobilized onto a SPR sensorprism CMD 200M chip (XanTec Bioanalytics, Germany) using a Continuous Flow Microspotter (Carterra, Dublin, CA, USA) to create an array of Abs. Kinetics and binning experiments were carried at 25 °C in a running buffer composed of 10 mM HEPES, pH 7.4, 150 mM NaCl, 3 mM EDTA and 0.005% Tween 20 (HBS-TE). The IBIS MX96 SPRi (Carterra, Dublin, CA, USA) was used to evaluate binding of hIL-33 to the immobilized Abs. For kinetics analysis, concentration series of reduced and oxidized hIL-33 starting at 300 nM was injected for 3 min (min) and allowed to disassociate for 10 min. The surface was regenerated between cycles with 10 mM Glycine pH 1.7. The binding data was processed using Scrubber (BioLogic Software). For Ab binning, reduced and oxidized hIL-33 was first injected for 4 min at 100 nM and was followed by a second 4 min injection of purified Ab at 10 µg/mL in a HBS-TE running buffer. The surface was regenerated between cycles with 10 mM Glycine pH 1.7. The binding data was processed using Epitope Binning software tool (Carterra, Dublin, CA, USA).

### Characterization of anti-hIL-33 Abs by SPR

A Series S Protein A sensor chip (Cytiva, Marlborough, MA, USA) was used to capture in-house recombinant chimeric human IgG versions of anti-hIL-33 Abs using a Biacore 8k instrument (Cytiva, Marlborough, MA, USA). Ab binding to human histidine-tagged hIL-33 (Genentech, South San Francisco, CA, USA) was measured using multi-cycle kinetics. Sensorgrams were recorded using an injection time of 2 min with a flow rate of 30 µL/min, at 25 °C or 37 °C, and with a running buffer of 10 mM *N*-2-hydroxyethylpiperazine-*Nʹ*-2-ethanesulfonic acid (HEPES), pH 7.4, 150 mM sodium chloride (NaCl), 3 mM ethylenediaminetetraacetic acid (EDTA), and 0.005% Tween 20. After injection, dissociation of hIL-33 from the Ab was monitored for 10 min in running buffer. The surface was regenerated between binding cycles with a 30 µL injection of 10 mM Glycine hydrochloric acid (HCl) pH 1.5. After subtraction of a blank containing running buffer only, sensorgrams were analyzed using a 1:1 Langmuir binding model with software supplied by the manufacturer to calculate the kinetics and binding constants.

### Reduced hIL-33 ELISA

MaxiSorp plates (384-well, Nunc, Thermo Fisher Scientific, Rochester, NY, USA) were coated with 1 µg/mL rat IgG2a anti-human hIL-33 15.21C7 Ab (Genentech, South San Francisco, CA, USA) in PBS, pH 7.4 overnight at 4 °C. Plates were then washed with 0.05% Tween 20 in PBS, pH 7.4, and blocked with 0.5% bovine serum albumin (BSA), 15 ppm ProClin 300 in PBS, pH 7.4 for 1 h at RT. Reduced hIL-33 standard was stored in 1 mM TCEP to maintain the protein in its reduced form and samples were diluted using a two-and-a-half step dilution in ice-cold assay buffer (0.5% BSA, 0.05% Tween 20, 15 ppm ProClin, 0.25% CHAPS, 5 mM EDTA, and 0.35 N NaCl in PBS, pH 7.4) and incubated in the plate for 2 h at RT. After 6 washes, 200 ng/mL biotinylated rat/human chimeric anti-hIL-33 3F10 MAb (Genentech, South San Francisco, CA, USA) diluted in 0.5% BSA, 0.05% Tween 20, 15 ppm ProClin in PBS, pH 7.4 was incubated for 1 h at RT. Bound reduced hIL-33 was detected using 1:10,000 dilution of HRP-conjugated streptavidin (GE Healthcare Life Sciences, Pittsburgh, PA, USA) and its substrate 3,30,5,50-tetramethyl benzidine (Moss Inc., Pasadena, MD, USA). The reaction was stopped with 1M phosphoric acid and absorbance was read at 450 nm. The titration curves were fitted using an in-house, 4-parameter regression program.

### Total hIL-33 ELISA

Total hIL-33 ELISA was carried out similarly using a 1:1 volume to volume ratio (vol/vol) mixture of 0.5 µg/mL rat IgG2a anti-hIL-33 15.21C7 (Genentech, South San Francisco, CA, USA) and 2 µg/mL goat anti-hIL-33 Abs (R&D Systems, Minneapolis, MN, USA) for coat and 1:1 (vol/vol) mixture of 100 ng/mL biotinylated goat anti-huST2 (R&D Systems, Minneapolis, MN, USA) and 50 ng/mL biotinylated goat anti-hIL-33 (R&D Systems, Minneapolis, MN, USA) Abs for detection. The standards consisted of a 1:1 (vol/vol) mixture of oxidized and reduced hIL-33 prepared in ice-cold assay buffer containing 20 ng/mL of hsST2.

### Ab-DNA conjugation

A 55-mer DNA 5ʹ-NH2-/5AmMC6/TGAAGGTCCTTGGCGATCATTTCGGCGGTGATCGGATGCATTTGGCTACGTCCCT-3ʹ (Integrated DNA Technologies, Coralville, IA) was activated with *N*-succinimidyl-(*N*-maleimidomethyl)cyclohexane-1-carboxylate (Pierce, Rockford, IL, USA). Rat IgG2a anti-human IL-33 clone 15.21C7 (Genentech, South San Francisco, CA, USA) was activated with *N*-succinimidyl-S-acetylthioacetate (Pierce, Rockford, IL, USA) and conjugated to the activated deoxyribonucleic acid (DNA) following manufacturer’s instructions. Free DNA was removed using a Superose 12 gel filtration column. Free Ab was removed using a Vivapure Q mini column (Sartorius Stedim Biotech GmbH, Goettingen, Germany).

### 384-well iPCR method to detect reduced hIL-33

0.2 µg/mL biotinylated rat/human chimeric anti-hIL-33 3F10 capture Ab, standards or samples, and 25 ng/mL DNA-labeled rat IgG2a anti-hIL-33 15.21C7 detection Ab were preincubated in sample buffer (0.5% BSA, 0.05% Tween 20, 15 ppm ProClin, 0.25% 3-((3-cholamidopropyl) dimethylammonio)-1-propanesulfonate (CHAPS), 5 mM EDTA, 0.35 N NaCl and 250 μg/mL calf thymus DNA in PBS, pH 7.4) for 2 h at room temperature. 250 µg/mL calf thymus DNA (Sigma, St. Louis, MO, USA) was heated to 95 °C for 10 min and then kept on ice for at least 5 min prior to addition to the assay buffer. Reduced hIL-33 (Genentech, South San Francisco, CA, USA) in 1 mM TCEP was used as the standard and prepared on ice to decrease oxidation. Reduced hIL-33 concentrations in human VH, AH, serum, and NF samples were prepared on ice using sample buffer. A streptavidin-coated 384-well real-time PCR plate (Roche Applied Science-Custom Biotech, Penzberg, Germany) was blocked for 2 h with SuperBlock buffer (ThermoFisher scientific, Waltham, MA, USA) and washed 3 times with iPCR wash buffer (0.5% BSA, 0.05% Tween 20, 15 ppm Proclin in PBS, pH 7.4) using a 384-well plate washer (BioTek, Winooski, VT, USA). Ab–antigen mixture was then added to the plate, incubated for 20 min prior to washing 13 times with iPCR wash buffer. A 60X PCR mixture containing primer (5ʹ‐TGAAGGTCCTTGGCGATCA and 5ʹ‐AGGGACGTAGCCAAATGCAT) and probe (5ʹ-TTCGGCGGTGATCGp-3ʹ, labeled with a fluorescein dye and a quencher) was diluted to 1× in Universal Taqman Master Mix II (Applied Biosystems, Foster City, CA, USA) and PCR grade water before being added to the plate. Real-time PCR was carried out using a ViiA 7 Real-Time PCR system (Applied Biosystems, Foster City, CA, USA). The thermal cycling conditions were 95 °C for 10 min followed by 40 cycles of 95 °C for 15 s and 60 °C for 1 min. The standard curve was generated using an in-house, 4-parameter regression curve-fitting program.

### Human biological samples

VH and AH samples from control and acute AMD postmortem donors were purchased from Lions Eye Institute for Transplant and Research (Tampa, FL, USA) or BioIVT (Westbury, NY, USA). Note that the stage (early, intermediate, advanced) or the type of the disease (wet versus dry AMD) was not disclosed in the sample sheet provided by the vendors. Serum, NF, and plasma samples from control and “self-reported” asthmatic patients were acquired from Genentech Healthy Services (South San Francisco, CA, USA).

### Statistical analysis

Statistical tests were conducted using either an unpaired two-tailed Student’s t-test or a Mann–Whitney test. Error bars depict the standard deviation. A P-value < 0.05 was considered statistically significant.

## Supplementary Information

**Additional file 1: Figure S1.** hsST2 concentrations determined in VH (n = 22 samples) and serum (n = 53 samples) from control donors.

**Additional file 2: Figure S2.** Reduced and oxidized hIL-33 QC (A) SDS-PAGE gel analysis of hIL-33 diluted in either PBS (a) or 60% IMDM media (b) and incubated at 37 °C. Shown are samples taken at t = 0 and t = 18 h time intervals. The protein samples were run under non-reducing conditions and reducing conditions with dithiothreitol (DTT). Molecular weight standards were applied in lanes 3, 6 and 9. (B) Mass spectrometry analysis of hIL-33 incubated at 37 °C in (a) PBS at t = 0, (b) PBS at t = 18 h and (c) IMDM media at t = 18 h. The detected MW’s for hIL-33 in each spectrum are shown. The theoretical MW of hIL-33 with all of its 4 cysteine residues fully reduced is 19857.19 dalton (Da). For a fully oxidized hIL-33, the theoretical molecular weight (MW) with all 4 cysteines forming two intra-disulfide bonds is 19853.16 Da. Panels (a) and (b) show hIL-33 with its cysteine residues in a fully reduced state while panel (c) shows the hIL-33’s cysteine residues in an oxidized state.

**Additional file 3: Figure S3.** Binding kinetics of anti-hIL-33 Abs as determined by Biacore. Multi-cycle kinetics sensorgrams for hIL-33 binding to captured rat anti-hIL-33 15.21C7 MAb at 25 °C (A) and rat/human chimeric anti-hIL-33 3F10 MAb at 25 °C (B). (C) Kinetics constants for anti-hIL-33 Abs binding to reduced hIL-33 at 25.

**Additional file 4: Table S1.** Dilution linearity of total hIL-33 endogenously expressed in human matrices.

**Additional file 5: Figure S4.** Total hIL-33 concentrations in serum, plasma, and NF samples measured using the reduced hIL-33 iPCR assay. (A–C) Total hIL-33 concentrations measured in human NF (A), serum (B) and plasma samples (C) from nine control and nine asthma patients. ns = no significance. Data are means of duplicates or quadruplicates.

**Additional file 6: Figure S5.** HsST2 concentrations in serum (A) and NF (B) from nine control and nine asthma patients. ns = no significance. Data are means of quadruplicates.

## Data Availability

All data generated or analyzed during this study are included in this published article (and its additional information files).

## Abbreviations

Abs: Antibodies
AH: Aqueous humor
AMD: Age-related macular degeneration
BSA: Bovine serum albumin
CHAPS: 3-((3-Cholamidopropyl) dimethylammonio)-1-propanesulfonate
CLE: Constant light exposure
CpG: Cytosine phosphodiester bond guanine
CV: Coefficient of variation
Da: Dalton
DNA: Deoxyribonucleic acid
DTT: Dithiothreitol
EBAA: Eye Bank Association of America
EDTA: Ethylenediaminetetraacetic acid
ELISA: Enzyme-linked immunosorbent assay
FDA: Food and Drug Administration
GA: Geographic atrophy
h: Hour
HCl: Hydrochloric acid
HEPES: *N*-2-Hydroxyethylpiperazine-*N*ʹ-2-ethanesulfonic acid
hIL-33: Human Interleukin 33
hsST2: Human soluble ST2
HTP: High throughput
HRP: Horseradish peroxidase
Iba1: Ionized calcium-binding adapter molecule 1
IgG: Immunoglobulin G
IL-1RAcP: IL-1 receptor accessory protein
IL1RL1: Interleukin 1 receptor-like 1
IMDM: Iscove’s Modified Dulbecco’s Medium
iPCR: Immune-polymerase chain reaction
IRB: Institutional Review Board
KD: Dissociation constant
LLOQ: Lower limit of quantitation
MAb: Monoclonal antibody
Min: Minute
MPL: Monophosphoryl-lipid
MRD: Minimum required dilution
NF: Nasosorption fluid
PBS: Phosphate buffered saline
QC: Quality control
Rb: Rabbit
RPE: Retinal pigment epithelium
RT: Room temperature
s.c.: Subcutaneous
SD: Standard deviation
SDS-PAGE: Sodium dodecyl sulfate polyacrylamide gel electrophoresis
sST2: Soluble Interleukin 1 Receptor Like 1
t: Time
TCEP: Tris(2-carboxyethyl)phosphine
Th: T helper
ULOQ: Upper limit of quantitation
VH: Vitreous humor
vol/vol: Volume to volume ratio
