# Supplementary figures and images for: Development of an ultra-sensitive human IL-33 biomarker assay for age-related macular degeneration and asthma drug development
Source: J Transl Med. 2021 Dec 20;19:517. doi: 10.1186/s12967-021-03189-3 (PMC8686655; doi:10.1186/s12967-021-03189-3)

## Slide 1
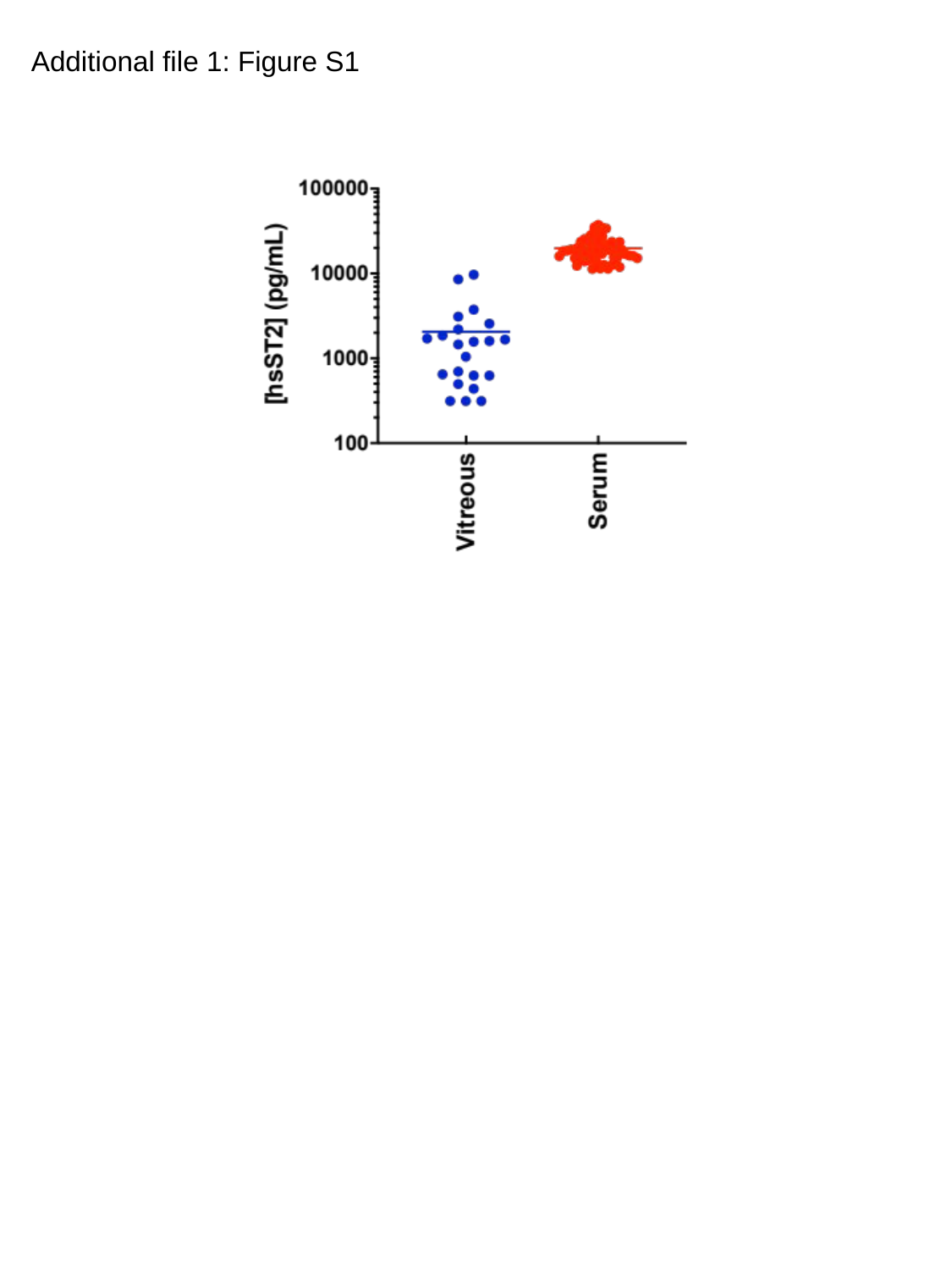

Additional file 1: Figure S1

Supplement: Supplementary file 1 — Additional file 1: Figure S1. hsST2 concentrations determined in VH (n = 22 samples) and serum (n = 53 samples) from control donors. [file 12967_2021_3189_MOESM1_ESM.pptx]

## Slide 1
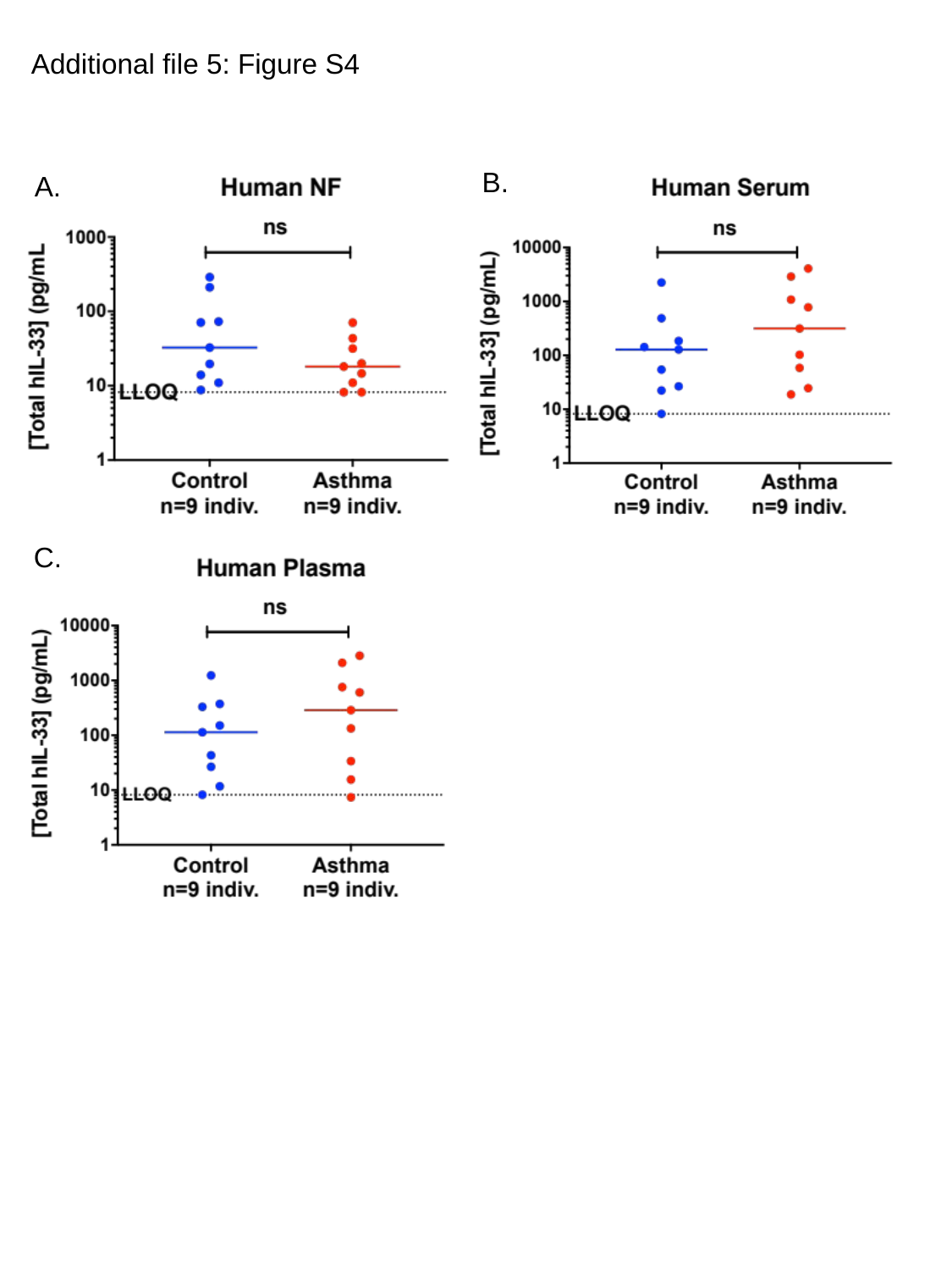

Additional file 5: Figure S4
B.
A.
C.

Supplement: Supplementary file 5 — Additional file 5: Figure S4. Total hIL-33 concentrations in serum, plasma, and NF samples measured using the reduced hIL-33 iPCR assay. (A–C) Total hIL-33 concentrations measured in human NF (A), serum (B) and plasma samples (C) from nine control and nine asthma patients. ns = no significance. Data are means of duplicates or quadruplicates. [file 12967_2021_3189_MOESM5_ESM.pptx]

## Slide 1
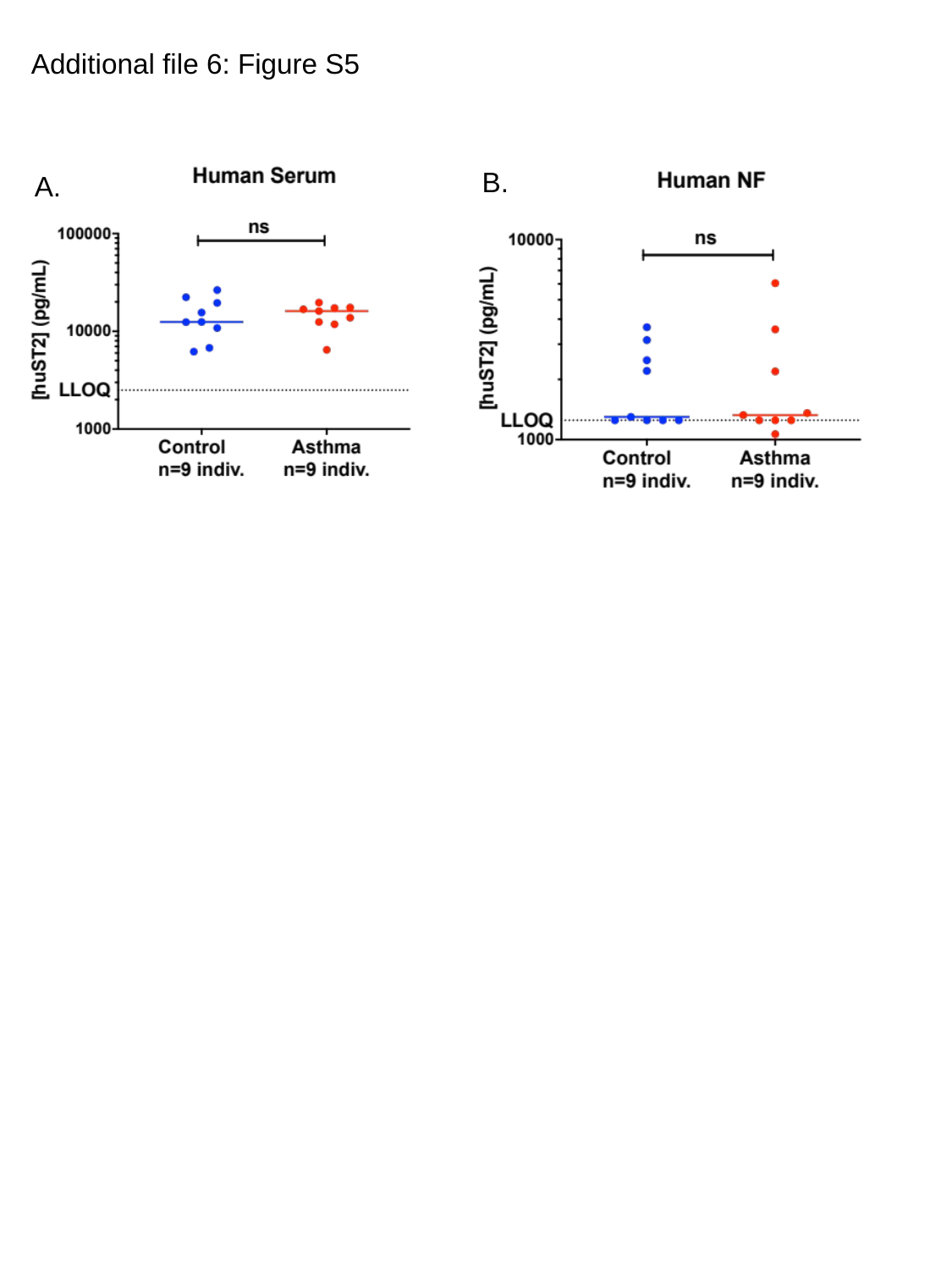

Additional file 6: Figure S5
B.
A.

Supplement: Supplementary file 6 — Additional file 6: Figure S5. HsST2 concentrations in serum (A) and NF (B) from nine control and nine asthma patients. ns = no significance. Data are means of quadruplicates. [file 12967_2021_3189_MOESM6_ESM.pptx]
