# Supplementary material for: Development of an ultra-sensitive human IL-33 biomarker assay for age-related macular degeneration and asthma drug development
Source: J Transl Med. 2021 Dec 20;19:517. doi: 10.1186/s12967-021-03189-3 (PMC8686655; doi:10.1186/s12967-021-03189-3)
Supplement: Supplementary file 2 — Additional file 2: Figure S2. Reduced and oxidized hIL-33 QC (A) SDS-PAGE gel analysis of hIL-33 diluted in either PBS (a) or 60% IMDM media (b) and incubated at 37 °C. Shown are samples taken at t = 0 and t = 18 h time intervals. The protein samples were run under non-reducing conditions and reducing conditions with dithiothreitol (DTT). Molecular weight standards were applied in lanes 3, 6 and 9. (B) Mass spectrometry analysis of hIL-33 incubated at 37 °C in (a) PBS at t = 0, (b) PBS at t = 18 h and (c) IMDM media at t = 18 h. The detected MW’s for hIL-33 in each spectrum are shown. The theoretical MW of hIL-33 with all of its 4 cysteine residues fully reduced is 19857.19 dalton (Da). For a fully oxidized hIL-33, the theoretical molecular weight (MW) with all 4 cysteines forming two intra-disulfide bonds is 19853.16 Da. Panels (a) and (b) show hIL-33 with its cysteine residues in a fully reduced state while panel (c) shows the hIL-33’s cysteine residues in an oxidized state. [file 12967_2021_3189_MOESM2_ESM.pptx]

## Slide 1
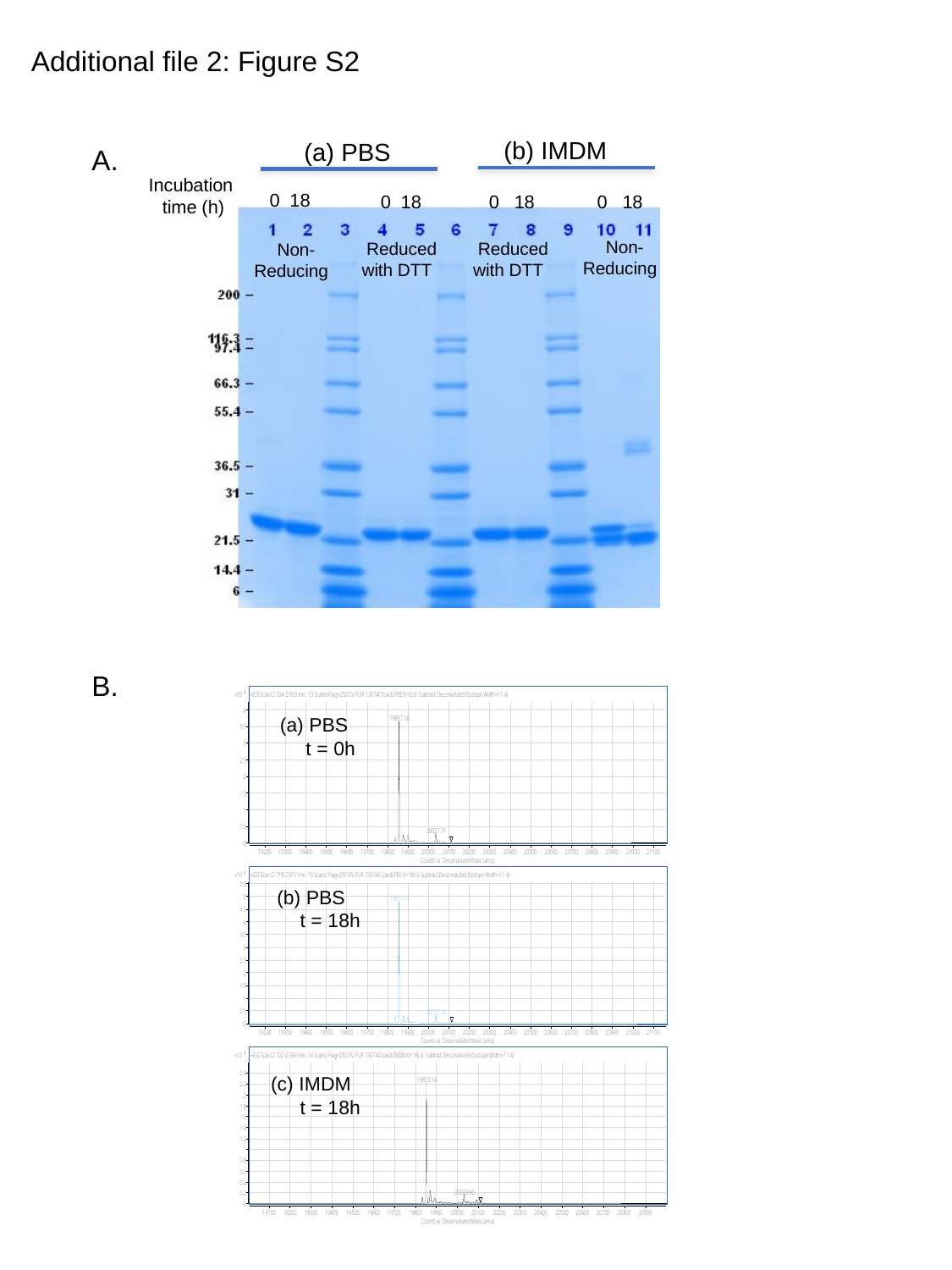

Additional file 2: Figure S2
(b) IMDM
(a) PBS
 0 18
 0 18
 0 18
 0 18
 Non-
Reducing
 Reduced
with DTT
 Reduced
with DTT
 Non-
Reducing
A.
Incubation
time (h)
B.
(a) PBS
 t = 0h
(b) PBS
 t = 18h
(c) IMDM
 t = 18h
