# Supplementary material for: Development of an ultra-sensitive human IL-33 biomarker assay for age-related macular degeneration and asthma drug development
Source: J Transl Med. 2021 Dec 20;19:517. doi: 10.1186/s12967-021-03189-3 (PMC8686655; doi:10.1186/s12967-021-03189-3)
Supplement: Supplementary file 3 — Additional file 3: Figure S3. Binding kinetics of anti-hIL-33 Abs as determined by Biacore. Multi-cycle kinetics sensorgrams for hIL-33 binding to captured rat anti-hIL-33 15.21C7 MAb at 25 °C (A) and rat/human chimeric anti-hIL-33 3F10 MAb at 25 °C (B). (C) Kinetics constants for anti-hIL-33 Abs binding to reduced hIL-33 at 25. [file 12967_2021_3189_MOESM3_ESM.pptx]

## Slide 1
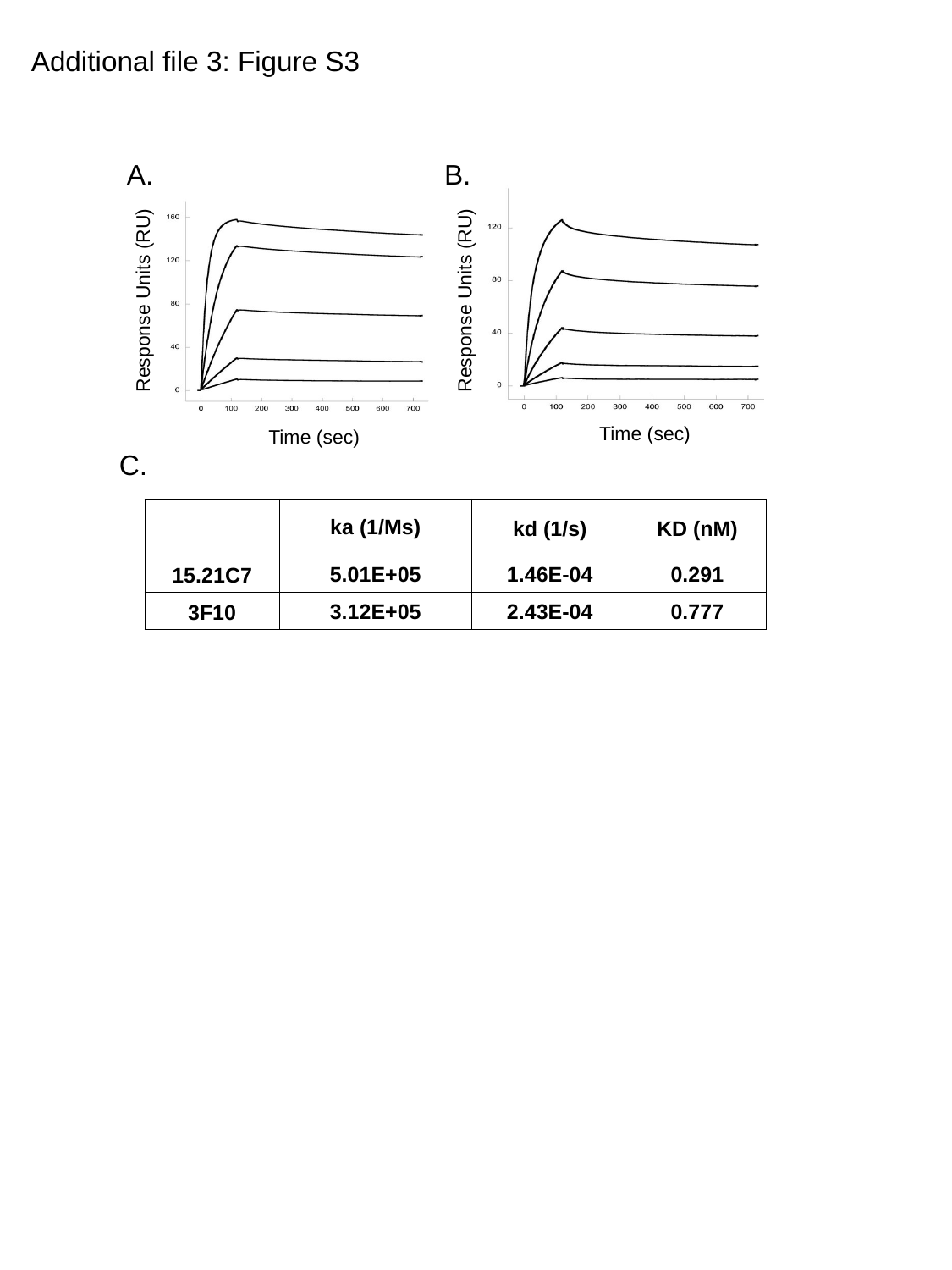

Additional file 3: Figure S3
A.
B.
Response Units (RU)
Response Units (RU)
Time (sec)
Time (sec)
C.
| | ka (1/Ms) | kd (1/s) | KD (nM) |
| --- | --- | --- | --- |
| 15.21C7 | 5.01E+05 | 1.46E-04 | 0.291 |
| 3F10 | 3.12E+05 | 2.43E-04 | 0.777 |
