# Supplementary material for: Development of an ultra-sensitive human IL-33 biomarker assay for age-related macular degeneration and asthma drug development
Source: J Transl Med. 2021 Dec 20;19:517. doi: 10.1186/s12967-021-03189-3 (PMC8686655; doi:10.1186/s12967-021-03189-3)
Supplement: Supplementary file 4 — Additional file 4: Table S1. Dilution linearity of total hIL-33 endogenously expressed in human matrices. [file 12967_2021_3189_MOESM4_ESM.pptx]

## Slide 1
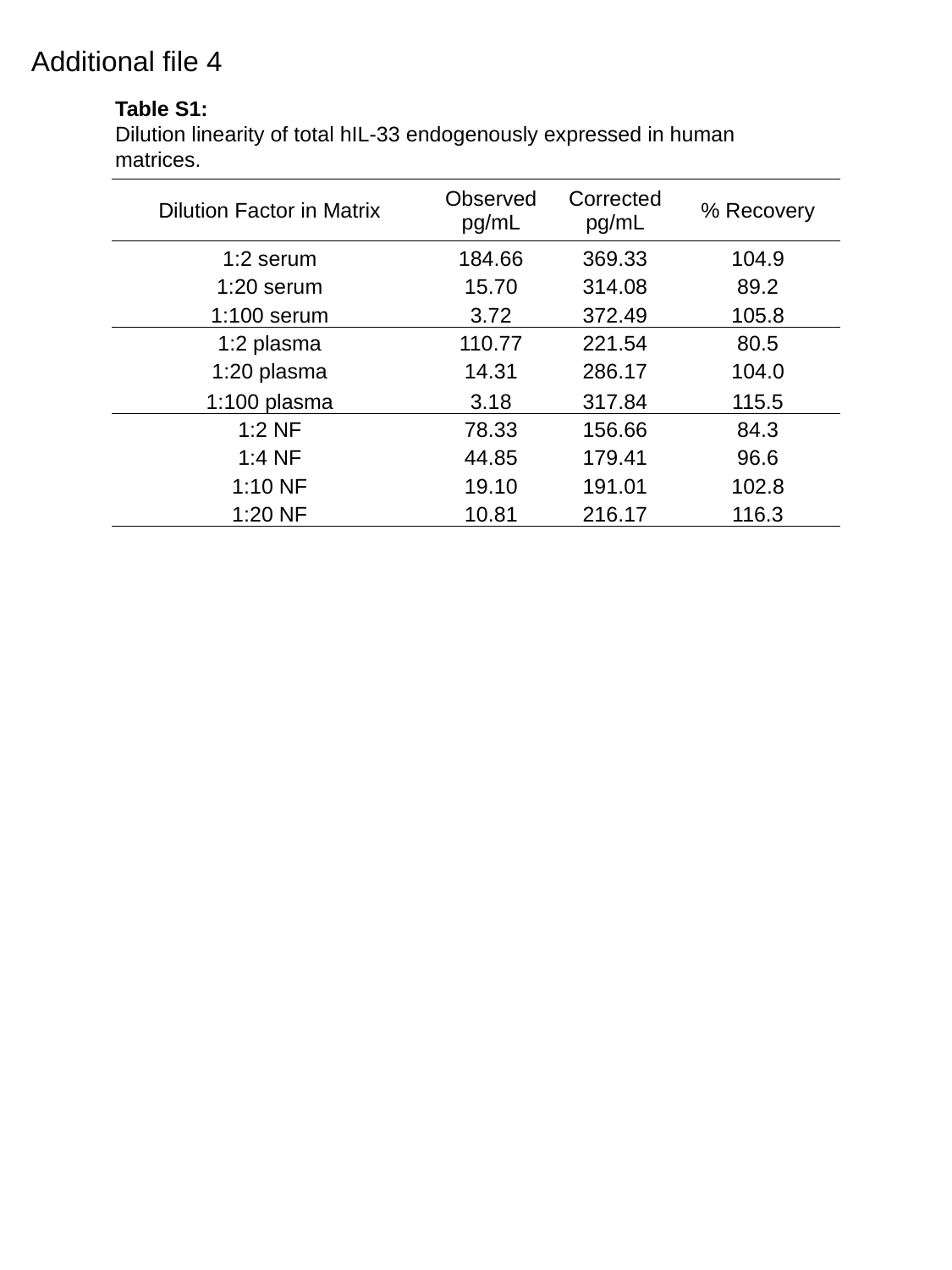

Additional file 4
Table S1:
Dilution linearity of total hIL-33 endogenously expressed in human matrices.
| Dilution Factor in Matrix | Observed pg/mL | Corrected pg/mL | % Recovery |
| --- | --- | --- | --- |
| 1:2 serum | 184.66 | 369.33 | 104.9 |
| 1:20 serum | 15.70 | 314.08 | 89.2 |
| 1:100 serum | 3.72 | 372.49 | 105.8 |
| 1:2 plasma | 110.77 | 221.54 | 80.5 |
| 1:20 plasma | 14.31 | 286.17 | 104.0 |
| 1:100 plasma | 3.18 | 317.84 | 115.5 |
| 1:2 NF | 78.33 | 156.66 | 84.3 |
| 1:4 NF | 44.85 | 179.41 | 96.6 |
| 1:10 NF | 19.10 | 191.01 | 102.8 |
| 1:20 NF | 10.81 | 216.17 | 116.3 |
